# The nonsteroidal anti-inflammatory drug sulindac reverses obesity-driven immunosuppression and triple-negative breast cancer progression

**DOI:** 10.1186/s13058-025-02134-2

**Published:** 2025-10-24

**Authors:** Michael F. Coleman, Shannon B. McDonell, Lydia K. Eisenbeis, Emily N. Devericks, Jobin Chandi, Om Dave, Jane B. Pearce, Sylvia Wang, Morgan Cody, Ximena M. Bustamante-Marin, Elaine M. Glenny, Erika T. Rezeli, Alyssa J. Cozzo, Ciara H. O’Flanagan, Brooke E. Bathon, Saame Raza Shaikh, Ginger L. Milne, Michael K. Wendt, Nadia A. Lanman, Dorothy Teegarden, Stephen D. Hursting

**Affiliations:** 1 Department of Nutrition, University of North Carolina, 235 Dauer Drive, MJHRC Room 2009, Chapel Hill, NC 27599 USA; 2 Lineberger Comprehensive Cancer Center, University of North Carolina, 235 Dauer Drive, MJHRC Room 2115, Chapel Hill, NC 27599 USA; 3 Department of Pharmacology, Vanderbilt University School of Medicine, Nashville, TN USA; 4 Department of Comparative Pathobiology, Purdue University, West Lafayette, IN 47907 USA; 5 Purdue University Institute for Cancer Research, Purdue University, West Lafayette, IN 47907 USA; 6 Nutrition Research Institute, University of North Carolina, Kannapolis, NC 28081 USA

**Keywords:** Obesity, Inflammation, TNBC, Antitumor immunity, Tumor microenvironment

## Abstract

Obesity affects over 40% of women in the US and increases the risk and progression of several cancers, including triple-negative breast cancer (TNBC), in part through chronic low-grade inflammation and impaired antitumor immunity. While weight loss can reverse obesity-driven cancer risk, cost and other factors limit the accessibility of effective weight loss interventions. This study investigated whether sulindac, a nonsteroidal anti-inflammatory drug (NSAID), could mitigate obesity-driven TNBC progression. Using multiple preclinical models, we demonstrate that sulindac treatment abrogates obesity-accelerated tumor growth and metastasis without affecting body weight or composition. Bulk transcriptomic profiling revealed obesity-driven suppression of immune-related gene signatures in the tumor microenvironment (TME)—including antigen presentation—while sulindac treatment restored these signatures. Single-cell RNA sequencing identified sulindac-mediated reprogramming of tumoral metabolism toward oxidative phosphorylation and restoration of antigen presentation machinery in tumor-associated macrophages. Sulindac also reversed obesity-driven reduction in T cell receptor diversity within the TME. We conclude that sulindac treatment remodels the TME and restores obesity-associated impairments of immunosurveillance, offering a potentially accessible intervention to limit obesity-driven TNBC progression. We demonstrate that NSAIDs, which are generally safe, cheap, and readily available, limit the burden of obesity-driven TNBC preclinically, warranting further evaluation as a targeted clinical intervention.

**Graphical abstract:**

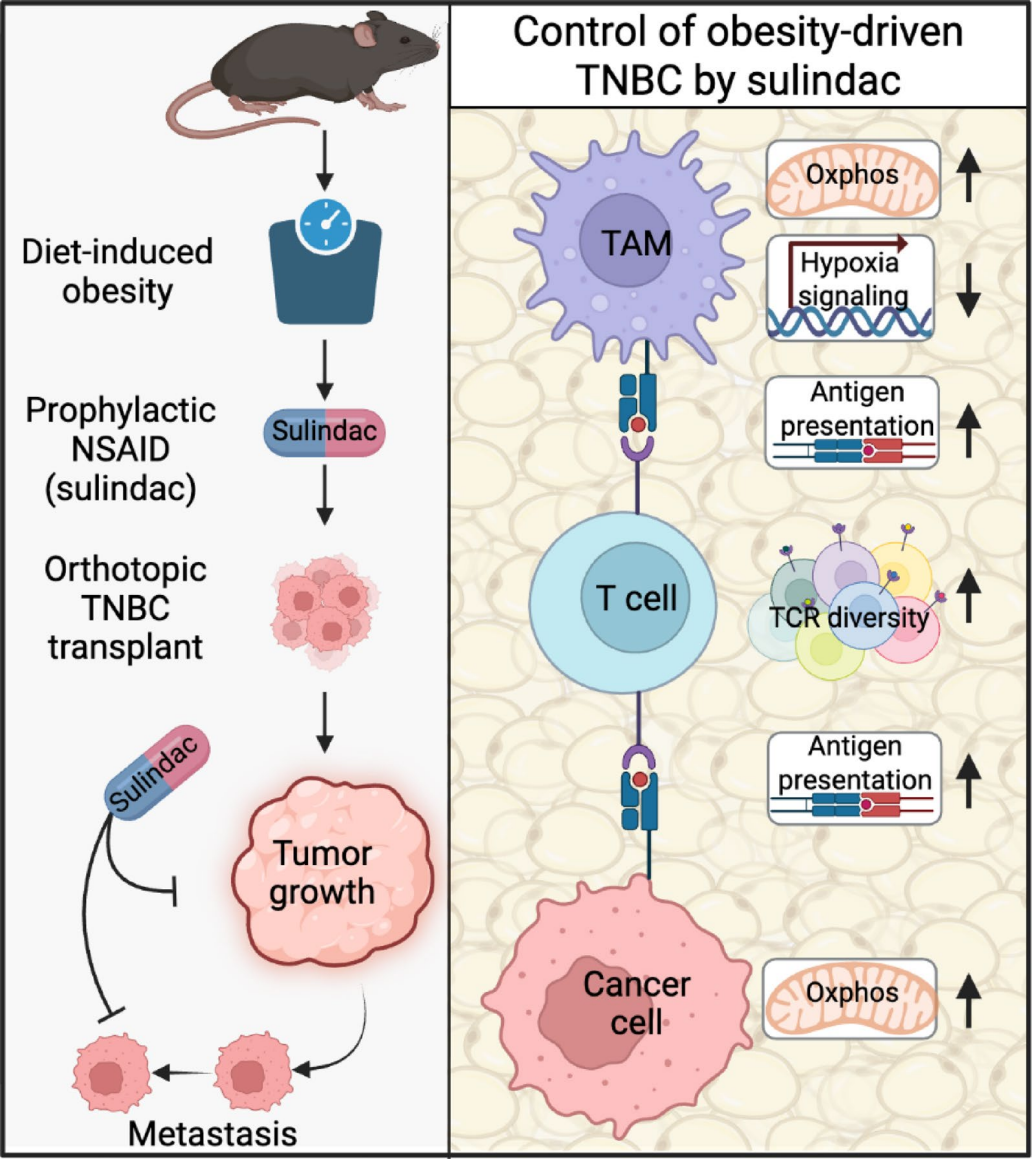

**Supplementary Information:**

The online version contains supplementary material available at 10.1186/s13058-025-02134-2.

## Introduction

Obesity, a disease that affects over 40% of US women [1], promotes the development and progression of several cancers including triple-negative breast cancer (TNBC) [2–4]. Metastatic TNBC (mTNBC) has markedly poor prognosis [3], and intervention strategies for controlling TNBC and its metastatic progression are urgently needed. Factors contributing to the increased risk of TNBC in patients with obesity include chronic low-grade inflammation, impaired immune function, and elevated growth factor signaling [3, 5]. Sustained weight loss reverses many obesity-associated metabolic and inflammatory changes in patients with obesity [6]. Sustained weight loss in women following bariatric surgery reduces the incidence of all cancers combined by > 30%, and obesity-driven cancers, including breast cancer, by > 40% [7, 8]. However, while bariatric surgery reliably induces weight loss and effectively treats various other obesity-associated comorbidities, cost, surgery-associated risk, and social factors make it inaccessible to most qualifying patients [9]. Similarly, modern incretin-mimetic drugs, such as semaglutide and tirzepatide, are effective treatments for obesity [10, 11] but high cost limits their accessibility, and their anticancer effects remain to be fully established. Additionally, while preclinical evidence holds promise that these pharmacotherapies blunt obesity-driven TNBC growth [12], epidemiologic or clinical evidence is lacking. Traditional lifestyle interventions are theoretically highly accessible and can promote weight loss. However, weight loss is typically modest and exceptionally difficult to maintain [13]. Thus, for most patients with obesity, there is a dearth of simple, accessible, and effective interventions to mitigate heightened TNBC risk.

TNBC progression is a complex process driven by intrinsic tumor cell properties, extrinsic signals, and immunoregulation within the tumor microenvironment [14]. In TNBC cancer cells, obesity-derived molecular cues promote epithelial-to-mesenchymal transition (EMT) [15], extracellular matrix remodeling [16], and the formation of metastasis-initiating cells [17], thereby accelerating tumor progression. Extrinsic to cancer cells one of the most potent checks on tumor progression is immunosurveillance in the TME mediated in part through the adaptive immune system. Evidence that obesity impairs the normal functioning of adaptive immunity first emerged in vaccine efficacy data [18] and has grown progressively [19]. Indeed, in obesity, chronic inflammation and metabolic perturbations limit the ability of T cells to control tumor growth, including TNBC growth. Numerous obesity-associated antitumor immunity defects have been implicated in impairment of antitumor immunity by obesity, including diminished tumor-associated macrophage (TAM) antigen presentation [20], altered TAM metabolism [21], and dysfunctional T cell metabolism [22, 23]. Preclinical evidence indicates that restoration of antitumor immunity is an important mechanism through which weight loss limits obesity-driven tumor growth [23–25].

Over the past several decades a capacious understanding of the role for inflammatory signaling in cancer has developed [26], with numerous investigators finding that systemic anti-inflammatory treatment using nonsteroidal anti-inflammatory drugs (NSAIDs) limits progression of breast, and other, cancers [27, 28]. Moreover, chronic low-grade inflammation is a hallmark of obesity that both promotes tumor progression and blunts antitumor immunity [29]. While inflammatory signals can have mixed effects on tumor initiation and growth, they are generally associated with increased metastatic spread. Oxylipins produced by COX1 and COX2 (the molecular targets of NSAIDs) mediate both obesity and tumor associated inflammation, and play critical roles in cancer progression by regulating inflammation and its resolution [19, 30, 31]. Additionally, in cancer cells many inflammatory signals, including IL6, TNF, and oxylipins [32, 33], directly regulate cellular metabolism [32, 33], further complicating the study of inflammation in cancer. Importantly, the presence of a tumor-extrinsic inflammatory insult frequently promotes tumor progression and is readily targetable by systemic anti-inflammatory treatments. For example, acute post-surgical inflammation promotes breast cancer metastasis and can be mitigated by treatment with NSAIDs [34].

Using single cell and bulk transcriptomic profiling of TNBC, we investigated how treatment with sulindac (a prototypical NSAID) limits obesity-driven progression of TNBC. We identify TME remodeling as key to the protective effects of sulindac. Specifically, sulindac restores markers of antitumor immunity while suppressing tumor growth and metastasis; enforcing a tumoral metabolic program that restores antigen presentation and T cell receptor (TCR) diversity suppressed by obesity (see graphical abstract).

## Results

### COX1/2 inhibition abrogates obesity-driven TNBC growth and metastasis

**Fig. 1:**
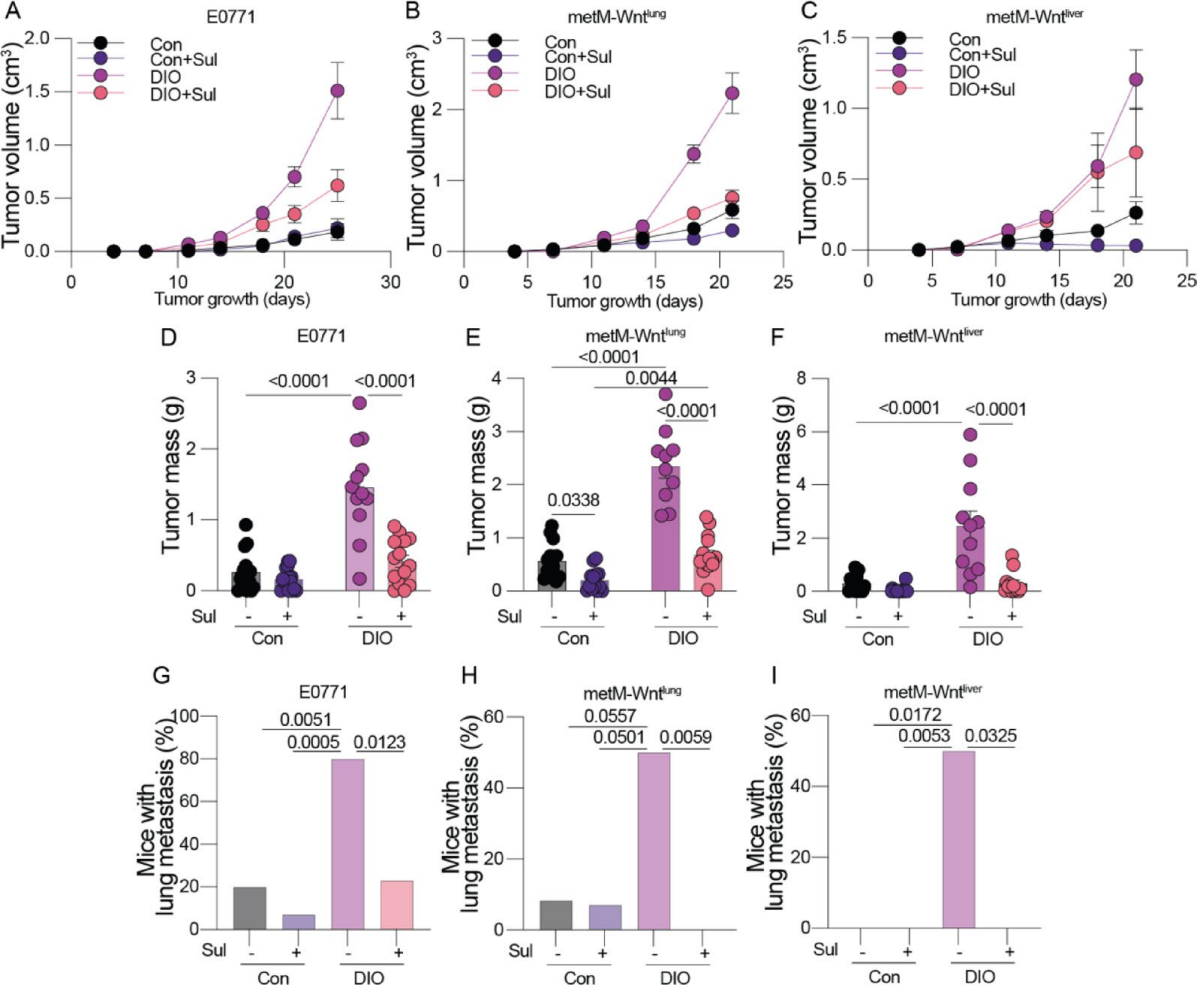
Sulindac suppresses obesity-driven TNBC growth and metastasis. Normoweight control (Con) and obese (DIO) mice treated with or without sulindac (Sul). Con, ConSul, DIO, and DIOSul mice were orthotopically injected with E0771 (n = 12–16/group), metM-Wnt^lung^ (n = 10–13/group), or metM-Wnt^liver^ (n = 11–20/group) cells. Mammary tumor growth over time of E0771 (**A**), metM-Wnt^lung^ (**B**), and metM-Wnt^liver^ (**C**) tumors. Mass of E0771 (**D**), metM-Wnt^lung^ (**E**), and metM-Wnt^liver^ (**F**) tumors. Percentage of mice with lungs containing micrometastases from E0771 (**G**), metM-Wnt^lung^ (**H**), and metM-Wnt^liver^ (**I**) tumors. Statistical significance determined by two-way ANOVA followed by Šídák's post hoc test (**D**–**F**) or Fisher’s exact test with posthoc correction for multiple hypothesis testing (**G**–**I**)

Obesity is well established to induce systemic inflammation, which consequently promotes TNBC growth and progression [5], which we and others have modeled effectively in vivo [22, 25, 35]. Hence, we sought to determine whether suppression of inflammation via treatment with an NSAID could limit TNBC primary tumor growth and metastatic spread in diet-induced obese (DIO) mice. We selected sulindac as our NSAID because it potently inhibits both COX1 and COX2 to block prostaglandin production. Normoweight control (Con) and DIO mice were treated with (ConSul and DIOSul) or without sulindac (140 ppm via diet) for 5 weeks, then orthotopically injected with one of 3 mTNBC cell lines (E0771, metM-Wnt^lung^, and metM-Wnt^liver^). Obesity accelerated TNBC progression, resulting in more rapid tumor growth and larger terminal tumor mass (Fig. 1A–F) and promoted lung metastasis (Fig. 1G–I) across all three TNBC cell lines, an effect which sulindac treatment consistently abrogated (see graphical abstract). We monitored body weight over the course of sulindac treatment and found sulindac to cause little to no weight loss, foreclosing the possibility that sulindac’s antitumor effects arouse due to weight loss or reduced metabolic impairment (Fig. S1A, B). Similarly, mammary fat pad and visceral adipose tissue mass were not reduced in sulindac-treated mice, indicating that the anticancer activity of sulindac was independent of weight loss or changes in body composition (Fig. S1C, D). COX1/2 potently regulate inflammation by directing intracellular production of prostaglandins. Therefore, we confirmed that sulindac treatment suppressed tumoral prostaglandin E_2_ (PGE_2_) levels in both control and DIO mice (Fig. S1E). Sulindac also suppressed PGF2, 6-keto PGF1ɑ, and 9,10-EpOMEs in DIO mice, which confirms that a drug concentration sufficient to limit COX activity was achieved within the TME (Fig. S1F–H).

### COX1/2 enables mTNBC cell migration

Metastatic progression of TNBC represents a distinct cascade of events wherein cancer cells migrate from the primary tumor, enter circulation, and subsequently establish colonies in distal sites. Migratory capacity and metabolic reprogramming are critical for this metastatic cascade [36]. To explore how tumor cell-intrinsic differences may drive growth and migratory capacity, we first turned to established in vitro murine paired, and metabolically distinct, nonmetastatic and metastatic TNBC cell lines to determine cellular underpinnings of metastatic capacity.

**Fig. 2:**
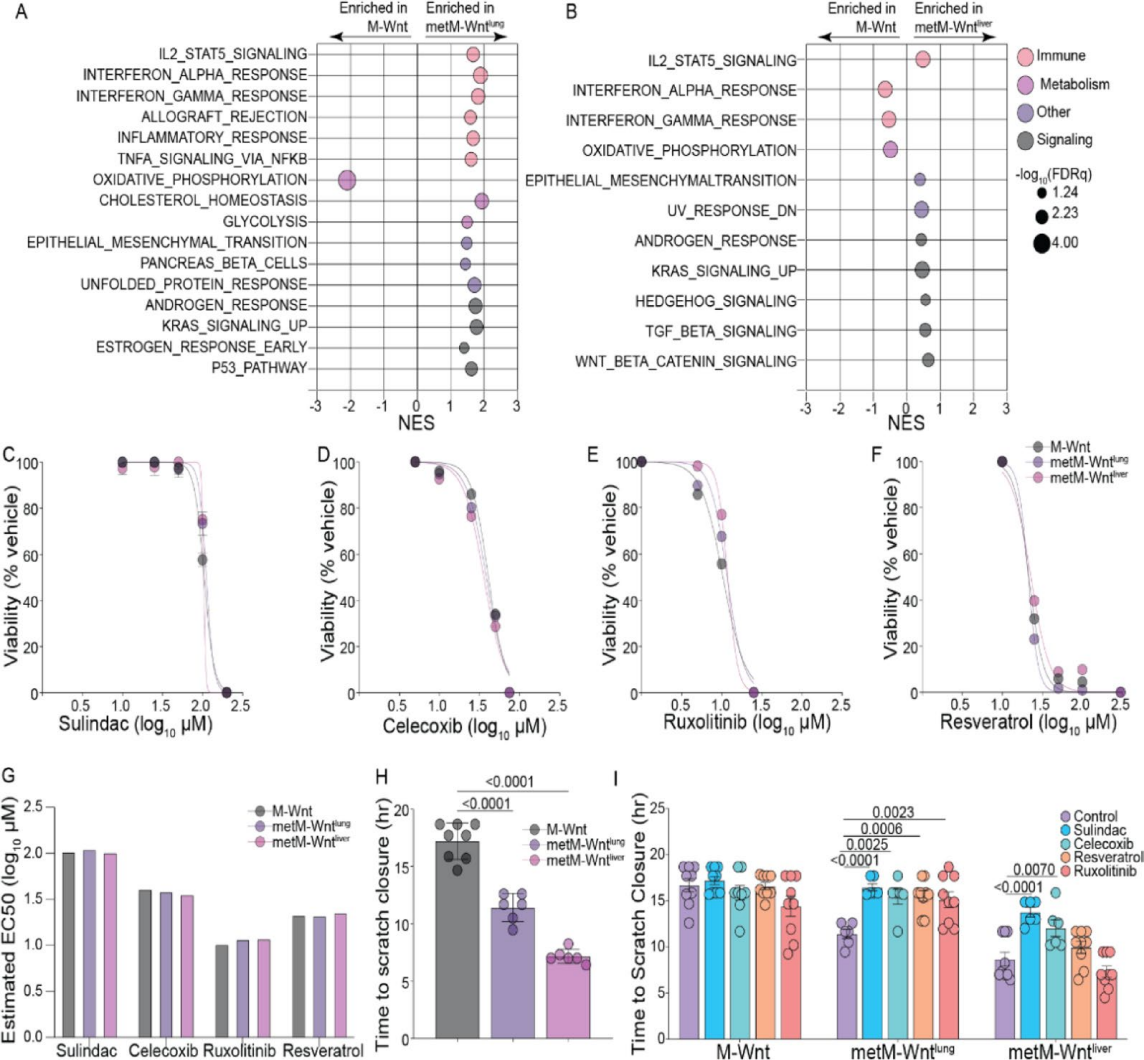
COX1/2 expression supports metastatic TNBC cell migration. Significantly enriched GSEA Hallmark gene sets in pairwise comparisons of in vitro transcriptomic profiles of M-Wnt with metM-Wnt^lung^ (**A**) or metM-Wnt^liver^ cells (**B**) (n = 3/group). Normalized enrichment scores (NES) and − log10 (FDRq) values are presented. Relative viability of M-Wnt, metM-Wnt^lung^, and metM-Wnt^liver^ cells treated with a range of concentrations of sulindac (**C**), celecoxib (**D**), ruxolitinib (**E**), or resveratrol (**F**) (n = 3/group). Estimated EC50 for each cell line for each anti-inflammatory drug (**G**). Wound healing assay using M-Wnt, metM-Wnt^lung^, and metM-Wnt^liver^ cells, either untreated (**H**) (n = 6–8/group) or treated with sublethal doses of sulindac, celecoxib, ruxolitinib, or resveratrol (**I**) (n = 5–9/group). Statistical significance was determined by nonlinear curve fitting (**C**–**F**) or one-way ANOVA (**H**, **I**). FDRq < 0.05 was considered significant

We compared the transcriptomic profile of cultured parental, nonmetastatic M-Wnt cells with metastatic subclone lines previously isolated from lung or liver metastatic lesions (metM-Wnt^lung^ and metM-Wnt^liver^, respectively) [35]. Using gene set enrichment analysis (GSEA), we found that relative to the nonmetastatic M-Wnt cells, both metastatic subclones displayed suppressed signatures of oxidative phosphorylation and enriched signatures of inflammation (Fig. 2A, B). IL-2-STAT5 signaling was consistently elevated in metastatic subclones relative to the nonmetastatic parental line, with numerous other inflammation-related gene sets elevated in metM-Wnt^lung^ cells relative to M-Wnt cells (Fig. 2A, B). We also found that relative to M-Wnt cells, both metastatic subclones expressed higher levels of COX1 (*Ptgs1*), while metM-Wnt^liver^ cells also had elevated COX2 (*Ptgs2*) expression (Fig. S2A, B).

To determine if inflammatory signaling in the metastatic subclones increased sensitivity to anti-inflammatory drugs, we assessed cell viability of M-Wnt, metM-Wnt^lung^, and metM-Wnt^liver^ cells when treated with sulindac (COX1 and COX2 inhibitor), celecoxib (selective COX2 inhibitor), ruxolitinib (JAK1/2 inhibitor that blocks STAT signaling), or resveratrol (NF-kB inhibitor). Dose–response testing demonstrate that all cells tested were sensitive to these anti-inflammatory drugs, with similar reductions in viability across doses for the metastatic subclones and the parent M-Wnt cells (Fig. 2C–G). We next sought to determine whether the migration of metastatic subclones, which was significantly faster than that of parental M-Wnt cells at baseline (Fig. 2H), might be blunted by treatment with sublethal concentrations of these anti-inflammatory drugs. We found that both sulindac and celecoxib suppressed the migration of metM-Wnt^lung^ and metM-Wnt^liver^ cells, while no anti-inflammatory drug altered the rate of migration of M-Wnt cells (Fig. 2I). Thus, in this model of mTNBC migratory capacity was driven by inflammatory signaling and could be suppressed by anti-inflammatory drugs.

### Lung colonization is not altered by either sulindac or obesity

**Fig. 3:**
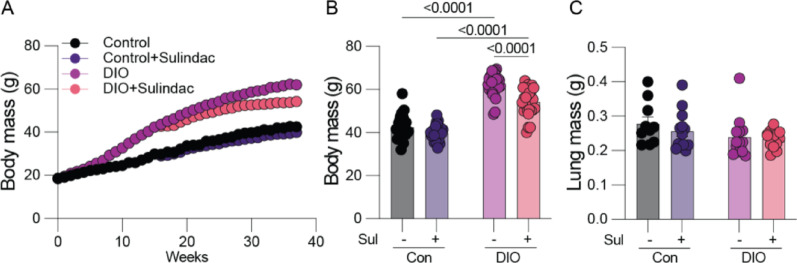
Sulindac and obesity do not alter lung colonization by TNBC cells. Normoweight control (Con) and obese (DIO) mice treated with or without sulindac (Sul). Con, ConSul, DIO, and DIOSul mice were injected with E0771 cells via tail vein and lung metastatic burden determined. Body mass over time (**A**), and terminal body mass (**B**) (n = 25–26/group). Terminal lung mass (**C**) (10–13/group). Statistical significance determined by two-way ANOVA followed by Šídák’s posthoc test (**A**–**C**)

Given our finding that sulindac suppressed metastatic cell migration and obesity-driven metastasis, we next sought to determine whether the protective effects of sulindac could be explained by alteration of the premetastatic lung microenvironment. Hence, we conducted a similarly designed experiment in which E0771 cells were delivered via tail vein, rather than orthotopic, injection to determine whether lung colonization was altered independent of changes early in the metastatic cascade (i.e., within the primary tumor). We employed E0771 cells as they are the most well-established model of mTNBC in C57BL6 mice. We assayed whether obesity or sulindac altered the efficiency of lung colonization by weighing the lungs ex vivo 28 days after injection. We found only limited changes in body weight by sulindac, and neither obesity nor sulindac treatment altered lung weight (Fig. 3A–C), indicating that in this model remodeling of the premetastatic niche within the lung was not a major determinant of either obesity-driven metastasis or sulindac-mediated protection.

### Sulindac restores markers of antitumor immunity that are suppressed by obesity

**Fig. 4:**
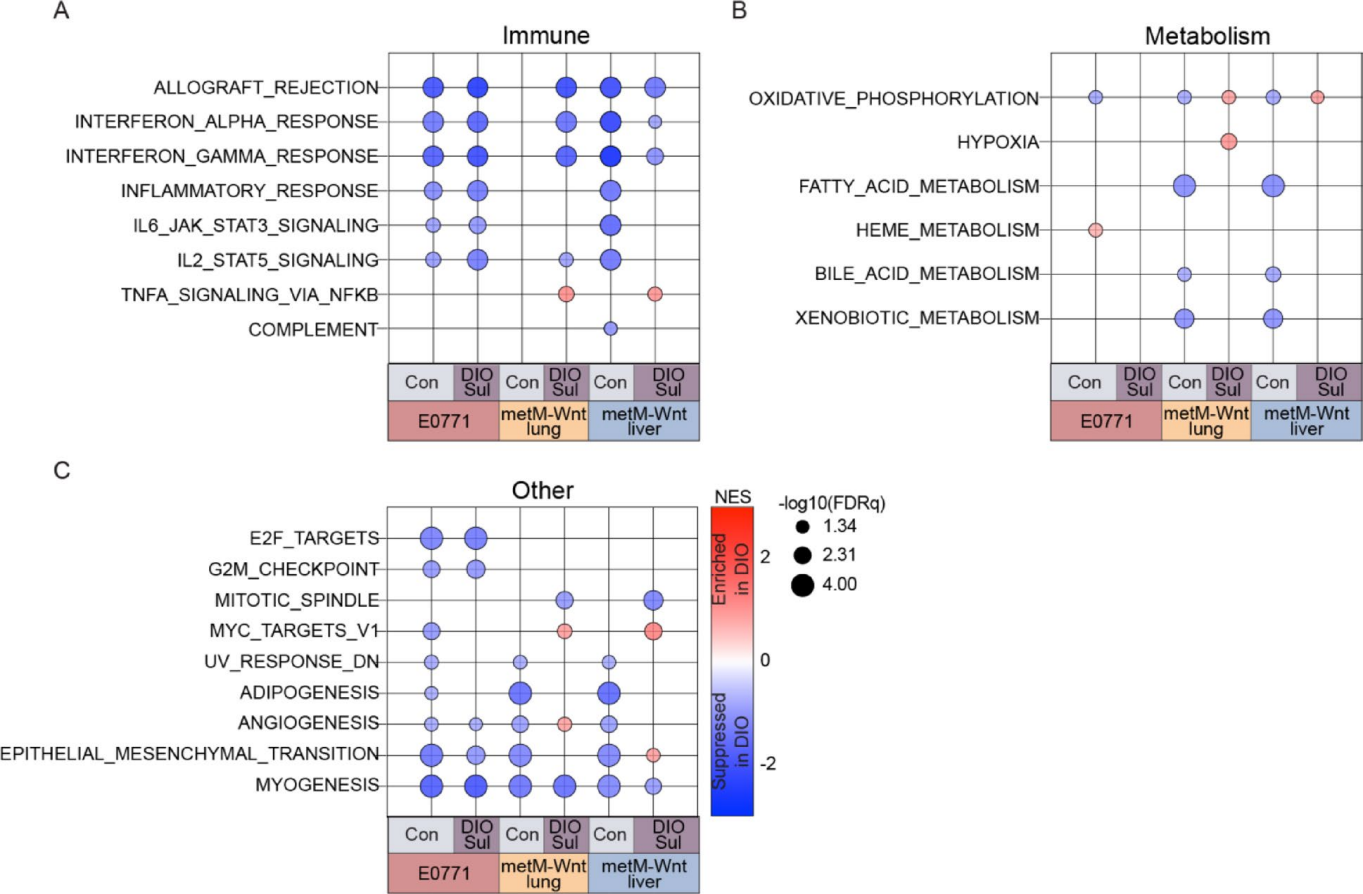
Sulindac reverses obesity-driven immunosuppression. Significantly enriched GSEA immune-related (**A**), metabolism-related (**B**), and other (**C**) Hallmark gene sets in pairwise comparisons of tumoral transcriptomic profiles of E0771, metM-Wnt^lung^, and metM-Wnt^liver^ primary tumors (n = 6–8/group). Transcriptomic profiles for tumors generated from each cell line were subjected to pairwise comparisons of DIO vs. Con and DIO vs. DIOSul. Normalized enrichment score (NES) and − log10 (FDRq) are presented. FDRq < 0.05 was considered significant

In light of the evidence that remodeling of the primary tumor was central to obesity-driven TNBC progression, we sought to define how obesity remodeled transcriptomic programs within the primary tumor. We performed transcriptomic profiling of mammary tumors induced by 3 mTNBC cell lines (E0771, metM-Wnt^lung^, metM-Wnt^liver^), and, using GSEA, identified numerous pathways and processes suppressed in tumors from DIO mice, relative to tumors from either control (Con) or sulindac-treated DIO (DIOSul) mice. Relative to Con or DIOSul mice, we found consistent suppression of immune-related gene sets in tumors derived from all 3 cell lines from DIO animals (Fig. 4A). Additionally, we found evidence that sulindac treatment reversed many of the metabolic and other perturbations induced in tumor cells by DIO (Fig. 4B, C). We performed a similar analysis of tumors from Con mice treated with sulindac (ConSul) and found that sulindac enhanced enrichment of immune- and metabolism-related, and other, gene sets in metM-Wnt^lung^ and metM-Wnt^liver^ tumors (Fig. S3A–C).

### Sulindac reprograms tumor cell metabolism to favor oxidative phosphorylation

To delineate how sulindac modulates the TME to protect against obesity-driven tumor growth, we performed scRNAseq on whole E0771 tumors from Con, ConSul, DIO, and DIOSul mice (Fig. S4A). We employed the E0771 model as it is well-established as obesity-responsive, exhibited the highly consistent restoration of obesity-driven defects by sulindac, and demonstrated limited response to sulindac in Con mice. Following quality control and unsupervised clustering, we sequentially subclustered the cells into cancer cells (*Ptprc*-negative, GFP-positive) (Fig. S4B), T cells (*Ptprc*-positive, *Cd3e*-positive, GFP-negative) (Fig. S4C), and myeloid cells (*Ptprc*-positive, *Cd3e*-negative, *Itgam*-positive, GFP-negative) (Fig. S4D). We then employed these subclustered analyses to define obesity-driven changes (i.e., DIO vs. Con) and assess whether sulindac reverses these changes (i.e., DIO vs. DIOSul).

**Fig. 5:**
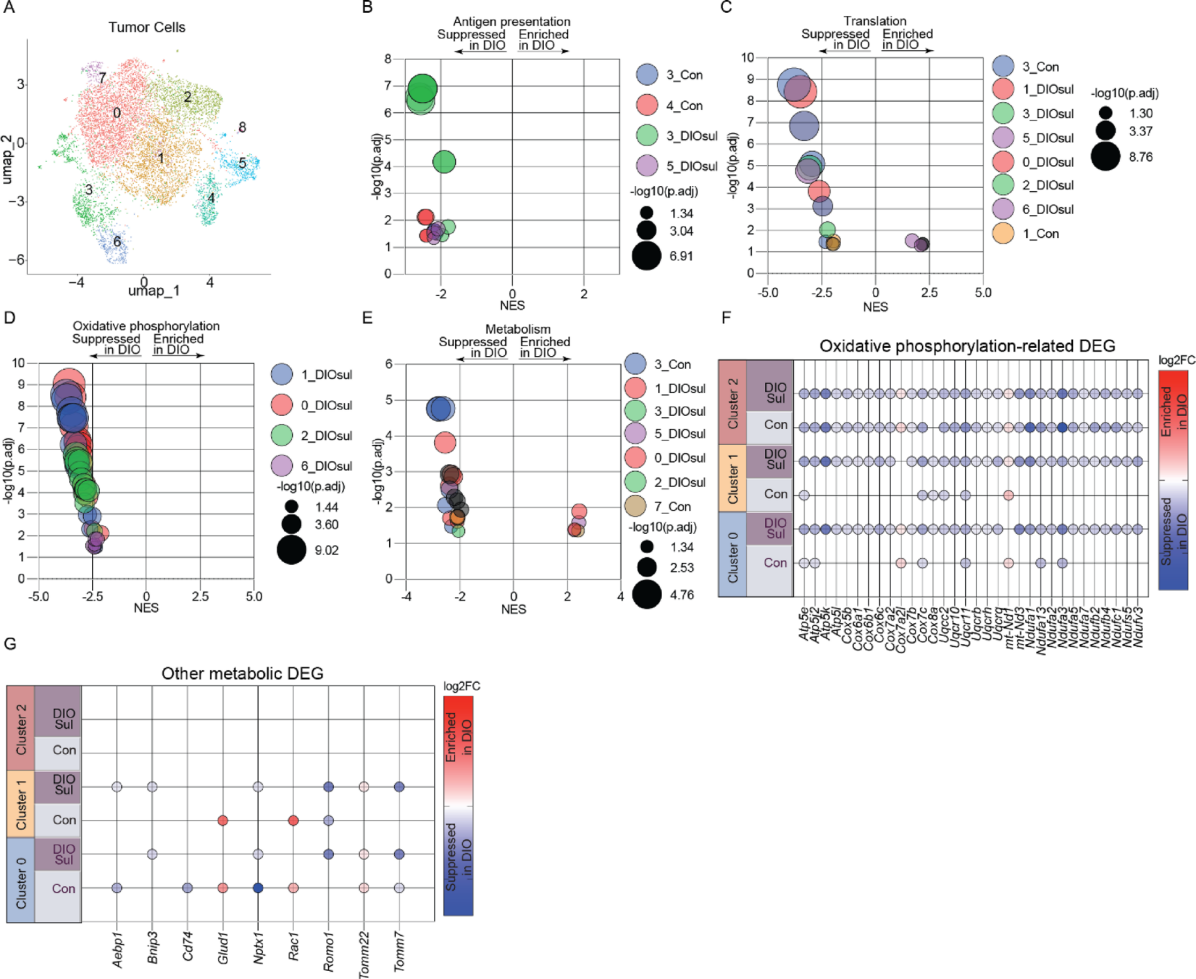
Sulindac reverses obesity-driven remodeling of tumor cells. scRNAseq of E0771 tumor cells subclustered based on GFP expression. UMAP projection (**A**). Significant GSEA enrichments generated, from indicated pathways, by cluster-wise comparison of DIO and DIOSul or Con groups. Antigen presentation (**B**), Translation (**C**), Oxidative phosphorylation (**D**), and Metabolism (**E**). Differentially expressed oxidative phosphorylation-related (**F**) and other metabolism-related (**G**) genes. Normalized enrichment score (NES) and − log10 (FDRq) are presented. FDRq < 0.05 was considered significant

Remodeling of cancer cell metabolism enables both metastatic spread and immunosuppression [37–39]. Given our observation that within bulk transcriptomic signatures sulindac appeared to reverse obesity-driven immunosuppression and metabolic perturbations in mTNBC tumors, we hypothesized that sulindac would enforce a metabolic program prohibitive of obesity-driven immunosuppression and metastasis. Hence, we first investigated obesity-driven changes within GFP-positive cancer cells by performing GSEA using Gene Ontology Bioprocesses on ranked gene lists from both DIO vs. Con and DIO vs. DIOSul in cluster-wise comparisons based on UMAP analysis (Fig. 5A). Numerous pathways in the GFP-positive cancer cells were suppressed by DIO relative to both Con and DIOSul, including those related to antigen presentation and protein translation (Fig. 5B, C). Consistent with sulindac-driven remodeling of cancer cell metabolism, cancer cells from DIO mice had marked suppression of oxidative phosphorylation-related gene sets relative to those from DIOSul mice. However, no enrichment of oxidative phosphorylation-related gene sets was evident between cancer cells from DIO and Con mice (Fig. 5D). Further, obesity-driven metabolic programming of cancer cells was evidenced by the marked differential enrichment of other metabolic gene sets (principally concerned with amide metabolism and biosynthetic reactions) between DIO and either the Con or DIOSul groups (Fig. 5E). To understand the molecular basis of the sulindac-driven enrichment of oxidative phosphorylation we profiled oxidative phosphorylation-related genes differentially expressed between DIO and Con or DIO and DIOSul. We found strikingly consistent enrichment of genes from complex I, III, IV, and V in multiple clusters from DIOSul, but not Con, relative to DIO (Fig. 5F). Similarly, we found several genes responsible for mitochondrial homeostasis to be differentially expressed across multiple clusters (*Bnip3*, *Romo1*, *Tomm22*, *Tomm7*) (Fig. 5G).

### Sulindac rescues obesity-related reduction of TCR diversity in tumor infiltrating T cells

**Fig. 6:**
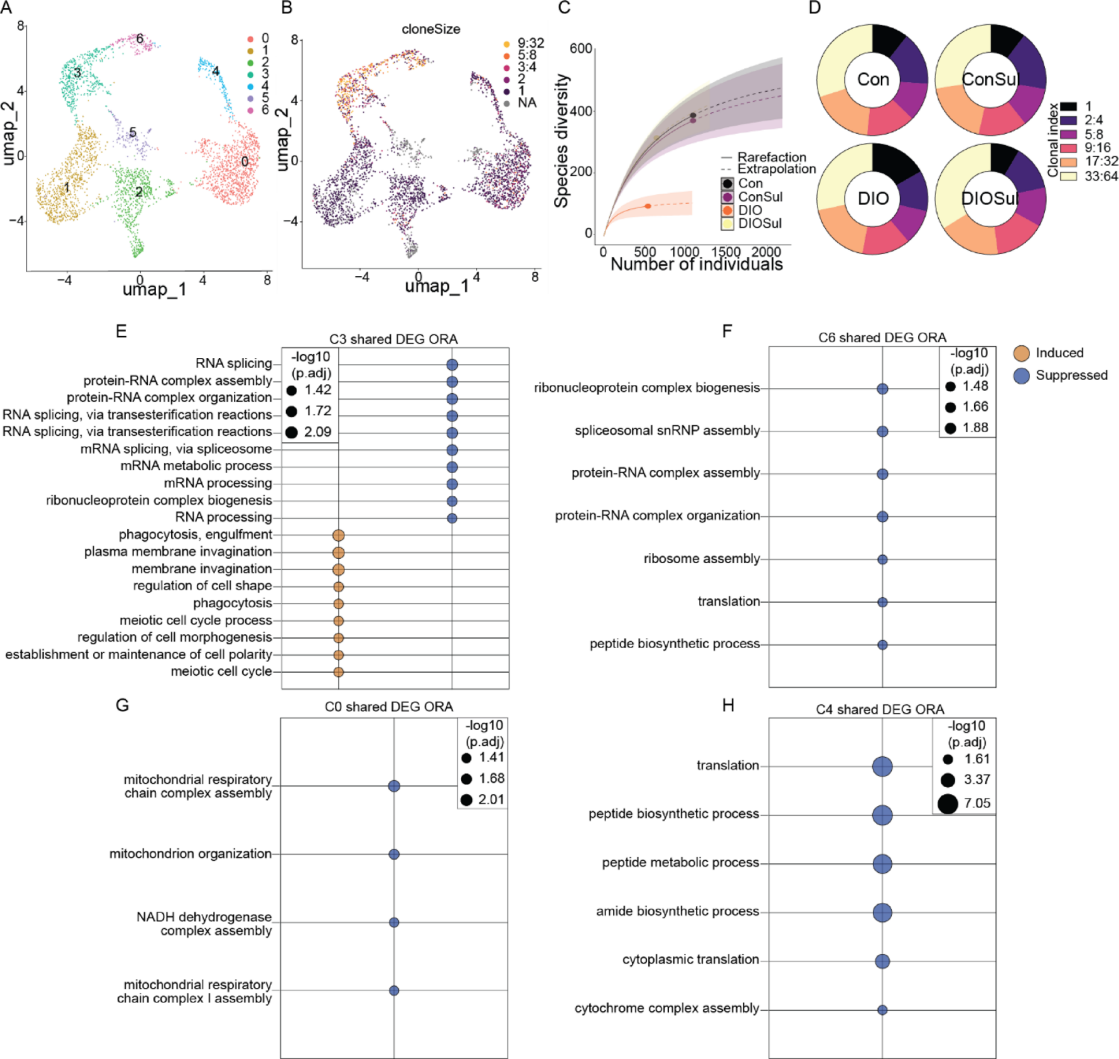
Sulindac restores TCR diversity to tumors of DIO mice. scRNAseq of T cells subclustered based on *Cd3e* expression. UMAP projection of global T cell population (**A**) and overlaid with TCR clone size (**B**). Rarefication plot of TCR diversity (**C**). Proportional abundance of TCR clonal indices (**D**). Cluster-wise over-representation analysis of DEG shared between comparisons of DIO and DIOSul, and DIO and Con groups (**E**–**H**). FDRq < 0.05 was considered significant

Blunted T cell mediated tumor control via perturbation of T cell metabolism is rapidly emerging as a key mechanism through which obesity promotes cancer [22, 23, 40]. Given that our bulk transcriptomic data demonstrated robust restoration of antitumor immunity by sulindac in obese mice, we next queried obesity-associated T cell dysfunction and the potential reversal of this dysfunction by sulindac. Specifically, to investigate how sulindac reverses obesity-driven immunosuppression, we first examined T cell subclusters (Fig. 6A). We performed TCR sequencing and overlayed TCR clonal abundance with UMAP analysis (Fig. 6B). We identified 2 major CD8+ T cell clusters of interest, both of which were clonally expanded and expressed exhaustion markers *Pdcd1* (Pd1), *Havcr2* (Tim3), and *Lag3* (C3 [terminally exhausted] and C6 [progenitor exhausted]) (Fig. 6B), while only C6 retained markers of active proliferation (*Mki67* and *Top2a*) (Fig. S5). Further, 2 Treg clusters (marked by *Cd4*+ *Foxp3*+) were identified (C4 and C0), of which only C4 expressed markers of proliferation (*Mki67* and *Topa2*) (Fig. S5). The remaining T cell clusters were considered bystander-T cells unlikely to be capable of mounting a significant antitumor response, and hence not further analyzed. To identify where clonal diversity was altered between T cells from tumors of each diet group, we analyzed the sequence diversity of TCRs in all T cells detected by rarefication plot. TCR diversity was markedly reduced in tumors from DIO mice relative to all other groups (Fig. 6C). Given the lower overall diversity of TCRs in DIO tumors, we next assessed whether the proportion of poorly, moderately, and highly expanded TCRs would differ between groups. To do so, we binned TCR clones by clonal index and found highly similar overall distribution of TCR clonalities, indicating that expansion of the TCRs present was not altered by either obesity or sulindac (Fig. 6D).

Metabolic stressors within the TME are known to profoundly limit T cell function in part by arresting T cell translation [41], yet how obesity-driven T cell dysfunction intersects with these observations is unclear. However, outside of the context of cancer, obesity-driven induction of ATF4 (a master regulator of protein translation) has been implicated in T cell dysfunction [42]. Thus, building on our observation that sulindac reversed obesity-driven impairment of TCR diversity, we hypothesized that mitigation by sulindac of obesity-driven metabolic abnormalities, including translation, would parallel restoration of T cell function. To test this hypothesis, we conducted differential tumoral gene expression analysis between DIO and either Con or DIOSul groups and then subjected the overlapping differentially expressed genes (DEG) from DIO vs. Con and DIO vs. DIOSul comparisons to overrepresentation analysis using Gene Ontology Bioprocesses. In both clonally expanded CD8 T cell clusters (C3 and C6) obesity suppressed signatures of translation (Fig. 6E, F). Within the Treg clusters (C0 and C4), obesity was again suppressive of numerous metabolic signatures, primarily affecting C0 oxidative phosphorylation signatures and C4 translation-related signatures (Fig. 6G, H).

### Sulindac reverses obesity-induced antigen presentation defects in TAMs

**Fig. 7:**
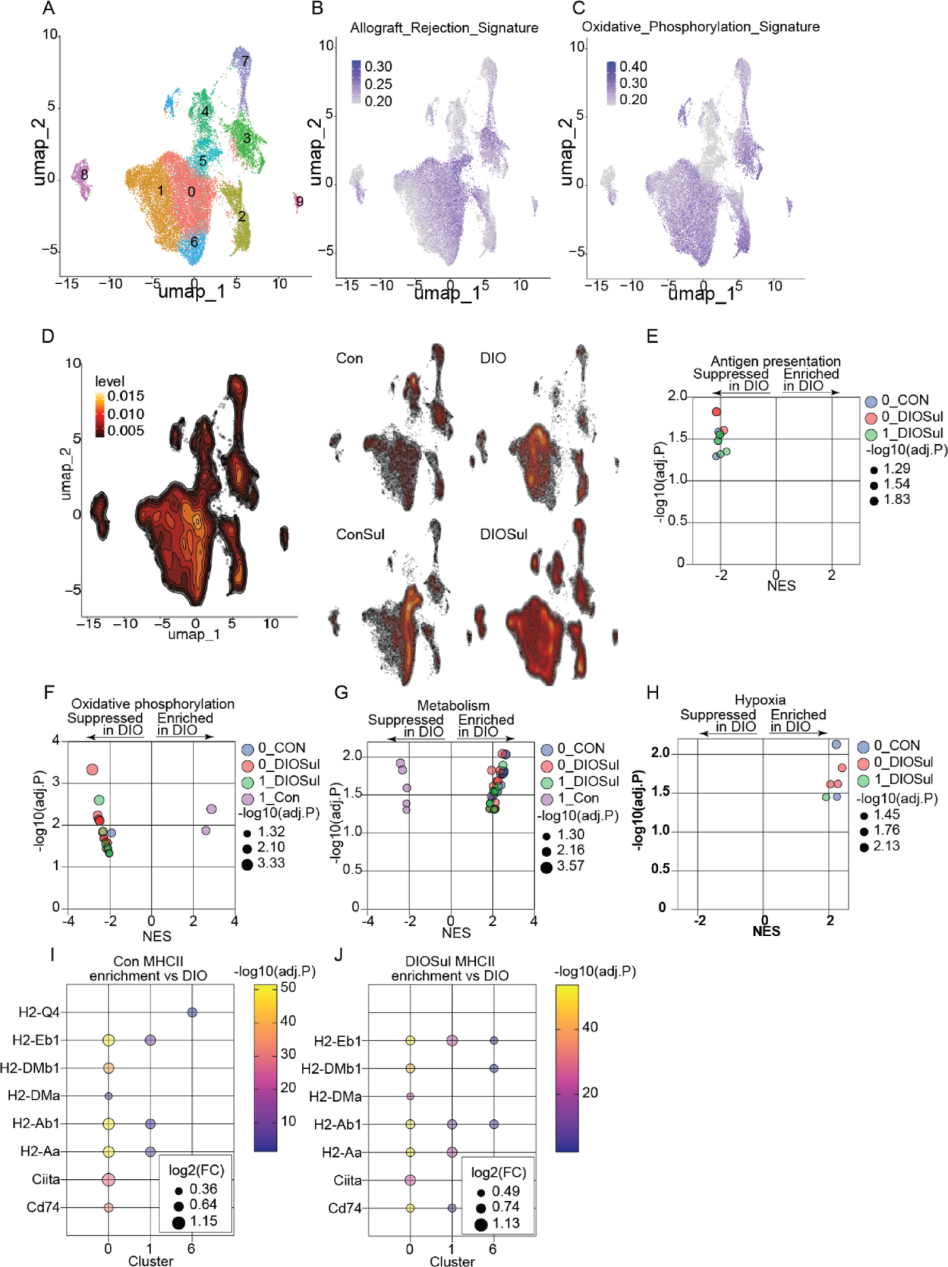
Sulindac restores antigen presentation and oxidative phosphorylation lost in TAMs from DIO mice. scRNAseq of myeloid cells subclustered based on *Itgam* expression. UMAP projection of global myeloid cell population (**A**) and overlaid with allograft rejection and oxidative phosphorylation signatures (**B**, **C**). Contour plot of cells from each group (**D**). Significant GSEA enrichments generated, from indicated pathways, by cluster-wise comparison of DIO and DIOSul or Con groups. Antigen presentation (**E**), oxidative phosphorylation (**F**), metabolism (**G**), and hypoxia (**H**). MHCII-related DEG from cluster-wise comparison of DIO and Con (**I**) or DIOSul (**J**) myeloid cells. Normalized enrichment score (NES) and − log10 (FDRq) are presented. FDRq < 0.05 was considered significant

Metabolism in TAMs regulates their activation and function, with obesity recently shown to suppress T cell function in part through suppression of antigen presentation by TAMs [20, 43, 44]. Thus, given the striking loss of TCR diversity in tumors from DIO mice (Fig. 6C), we next examined whether restoration of an obesity-driven impairment in antigen presentation underpins the ability of sulindac to normalize tumoral TCR diversity. To do so, we evaluated the myeloid subcluster within the scRNAseq analysis (Fig. 7A). Given the established links between obesity, metabolism, and TAM dysfunction [20, 21], we prioritized TAM populations within this cluster. We identified 3 major TAM clusters (C0, C1, C6) marked by *Itgam* (Cd11b), *Adgre1* (F4/80), *Ly6c2* (Ly6C), and *Ccr2* expression (Fig. S6A, B). To interrogate how the distribution of markers of cellular metabolism and antigen presentation within TAMs vary across groups, we performed UCell enrichment analysis using Hallmark gene sets for Allograft Rejection (Fig. 7B), Oxidative Phosphorylation (Fig. 7C), and Glycolysis (Fig. S6C). We then visually compared the density of an Allograft rejection signature in cells across groups (Fig. 7D). To more precisely quantify this observation, we performed GSEA analysis in a similar manner to Fig. 5. We found that DIO suppressed signatures of antigen presentation relative to Con and DIOSul in C0 (Fig. 7E), and that this suppression was coincident with DIO-driven suppression of markers of oxidative phosphorylation relative to Con and DIOSul (Fig. 7F). We next profiled the molecular basis of these oxidative phosphorylation-related enrichments. We found differentially expressed oxidative phosphorylation-related genes between DIO and Con or DIOSul to be comprised primarily of genes from complex I (*Ndufa13*, *Ndufa7*, *Ndufc1*), III (*Uqcr10*, *Uqcrb*, *Uqcrq*), and IV (*Cox5b*, *Cox6a1*, *Cox7b*) spanning TAM clusters from DIOSul and Con, relative to DIO (Fig. S7A). All but 2 of oxidative phosphorylation-related DEG overlapped with the differentially expressed oxidative phosphorylation DEG identified in tumors (Fig. S7B, C).

To further investigate how obesity remodels TAM metabolism, we examined the enrichment of a range of redox- and amino acid metabolism-related gene sets, and found their expression was generally enriched by DIO relative to Con and DIOSul (Fig. 7G). The exception was in TAMs from C1, where antigen presentation was not affected by DIO relative to Con (Fig. 7G). We profiled differential gene expression related to these metabolism-related enrichments. We found redox- (*Gpx4*, *Gsr*, *Prdx5*, *Prxl2b*) stress- (*Atf3*, *Oas1a*), and endoplasmic reticulum- (*Cln8*, *Dab2*, *Ehd1*) related genes to be differentially expressed between DIO and Con or DIOSul (Fig. S7D). Obesity promoted hypoxia related gene set enrichments in TAM clusters relative to both Con and DIOSul (Fig. 7H). To address whether obesity reduced expression of MHCII-related transcripts, we assayed differential TAM cluster gene expression between DIO versus either Con or DIOSul groups. We found consistent obesity-driven suppression of a range of these transcripts including CIITA, a master regulator of MHCII expression, in all TAM clusters relative to Con (Fig. 7I). Moreover, inhibition of COX1 and COX2 by sulindac fully reversed the obesity-driven suppression of antigen presentation machinery (Fig. 7J).

## Discussion

Obesity is second only to smoking as a preventable cause of cancer-related death in the US [45]. However, accessible and effective interventions to limit obesity-driven tumor progression remain underdeveloped. Herein, we demonstrate preclinical evidence that a prototypical NSAID disrupts obesity-driven tumor growth and metastatic spread via remodeling of the TME. Specifically, sulindac treatment blunts the ability of obesity to i) engender a metabolic program characterized by suppressed oxidative phosphorylation and dysregulated glycolytic metabolism in the TME, and ii) suppress TCR diversity and antigen presentation in TAMs. Critically, sulindac treatment achieves such important remodeling of the TME without any requirement for weight loss, and represents a class of widely used, well-tolerated, and readily available drugs.

Obesity has well-established immunosuppressive effects in the TME that culminate in limited T cell-mediated tumor control and loss of immune editing [23]. For example, obesity enforces a metabolic program in T cells within the TME that limits their capacity for tumor control [22, 40]. This T cell dysfunction in the TME has been attributed to obesity-driven alterations in circulating hormones and cytokines including leptin [46]. Similarly, elevated inflammatory signaling in obesity curtails antigen presentation by TAMs [21] in a PD1-dependent manner [20]. Here, we found that sulindac treatment reverses derangements in both the metabolic and antigen presentation pathways in the TME. While we and others have previously demonstrated that obesity-driven defects in antitumor immunity can be reversed via weight loss [23, 25, 47], we now show that sulindac, without inducing weight loss, restores the TCR repertoire lost in obesity.

Immunosuppression in the TME has been widely reported, with prior work demonstrating the potential for NSAIDs, through several mechanisms, to restore immunosurveillance to the TME [48, 49]. NSAIDs are typically systemically distributed following oral administration, and thus a wide range of cell types have been implicated in the restoration of antitumor immunity. For example, disruption of thromboxane A2 production in platelets by aspirin enables T cell-mediated tumor control [48]. Similarly, ibuprofen promotes macrophage differentiation, T cell function, and tumor control [49]. Intriguingly, NSAID use may also support immunotherapy response in lung cancers [50]. Beyond direct use of NSAIDs, targeted treatment with resolvins and prostaglandins [32] can achieve similar benefits. NSAIDs may be an effective adjuvant for immunotherapy, but the interaction with obesity has not been considered despite knowledge that obesity alters sensitivity to immune checkpoint inhibitors [51, 52]. We show that sulindac not only increases TCR diversity but also potently controls tumor growth. Thus, our preclinical findings suggest that NSAID use in patients with obesity and cancer may remodel the TME, which may be highly germane to immunotherapy use. We employed NSAID treatment prior to orthotopic tumor transplant to model prophylactic NSAID use in a population known to be at higher risk of TNBC. However, these protective effects observed remain to be validated in a clinical study, and cannot be generalized to NSAID initiation post-TNBC diagnosis.

Beyond immune-mediated control of primary tumor growth, control of metastatic spread is a key aspect of NSAIDs in cancer. Clinically, use of NSAIDs is associated with reduced rates of metastasis, and increased survival across numerous cancer types [53]. However, these effects are inconsistent across different patient cohorts. Preclinical evidence demonstrates that NSAIDs blunt metastatic spread of cancer directly induced by the acute inflammatory wound healing responses to surgery [34] or morphine use [54]. Similarly, disruption of thromboxane A2 production by aspirin blocks metastasis in melanoma and lung cancer [48]. We demonstrate that suppression of obesity-driven inflammation by sulindac blocks the acceleration of metastatic spread.

While, we and others have found that obesity suppressed transcriptomic signatures of oxidative phosphorylation in solid tumors [25, 55, 56], here we show sulindac consistently protected against obesity-mediated suppression of oxidative phosphorylation in multiple myeloid and tumor cell clusters. Obesity suppresses oxidative phosphorylation and antigen presentation in dendritic cells in colon cancer [56], which parallels the metabolic changes we observed in TAMs. TAM metabolism is generally characterized by elevated glycolysis and interrupted oxidative phosphorylation, superficially parallelling the metabolic phenotype of classically activated pro-inflammatory (M1 polarized) macrophages. Yet, activation of TAMs through CD40 promotes their antitumor activity of TAM by inducing, rather than suppressing, oxidative phosphorylation [57]. Similarly, TAMs engineered to sustain oxidative phosphorylation achieve enhanced inflammatory responses [58]. Thus, our finding that sulindac enhanced both oxidative phosphorylation and antigen presentation in tumors of obese mice may reflect the complex relationships between TAM metabolism, antitumor immunity, and obesity. Such an understanding does not, however, preclude the potential for partial antagonism between sulindac-driven metabolic changes and antitumor immunity. Similarly, the metabolic milieu of the TME in obesity, may be particularly sensitive to the protective effects of sulindac, as indicated by the limited protection offered by sulindac in Con mice.

Obesity remodels oxidative phosphorylation and fatty acid metabolism in numerous cell types within the TME including tumor cells [22, 25, 55, 56], and obesity-driven accumulation of fatty acids in the TME suppresses antigen presentation by TAMs [21]. We show sulindac enriched oxidative phosphorylation in multiple myeloid and tumor cell clusters from obese mice, yet suppresses oxidative phosphorylation in the transcriptomic profiles of whole tumors from obese, this apparent contradiction may reflect altered cellular activation states or composition of tumors between groups, or context specific effects of the cell lines used. However, we did observe enrichment of several electron transport chain genes (*Ndufa13*, *Ndufa7*, *Ndufc1*, *Uqcr10*, *Uqcrb*, *Uqcrq, Cox5b*, *Cox6a1*, *Cox7b)* which spanned multiple clusters from both myeloid and tumor cells.

In conclusion, our findings demonstrate that obesity significantly impacts TNBC tumor progression by modulating the tumor microenvironment. Moreover, we provide evidence that sulindac can restore antitumor immunity and reverse the procancer effects of obesity. These preclinical data offer a promising avenue for future clinical studies to assess the potential of sulindac or other NSAIDs to be used as an adjuvant therapy in women with TNBC and obesity.

## Methods

### Sex as a biological variable

Because breast cancers, and particularly TNBC, are very uncommon in men [59], only female mice were used in this study.

### Cell culture

E0771 mammary carcinoma cells were purchased from ATCC (CRL-3461, Manassas, VA) and transduced with GFP-NanoLUC. M-Wnt, metM-Wnt^lung^, and metM-Wnt^liver^ mammary carcinoma cells were developed in the Hursting laboratory from MMTV-Wnt1 mice on a C57Bl/6 background [35, 60]. All cell lines were maintained between 10 and 90% confluency in RPMI1640 media without phenol red (GIBCO Life Technologies) supplemented with 10% FBS, 10 mmol/L HEPES buffer, 2 mmol/L L-glutamine, and 1% penicillin/streptomycin and passaged with)0.05% trypsin EDTA. Cell lines were allowed to adhere for 24 h, then were treated with varying doses of sulindac (S3131, Millipore Sigma, Burlington, MA), celecoxib (S1261, SelleckChem, Houston, TX), resveratrol (S1396, SelleckChem), or ruxolitinib (S1378, SelleckChem). Following a 48-h incubation, viability was determined using CellTiter-Glo (Promega, Madison, WI), with the percent luminescence of vehicle treatment used as the reference. The highest dose that did not reduce viability (dose before exponential decline; 50 µM sulindac, 10 µM, celecoxib, 10 µM resveratrol, 5 µM ruxolitinib) was then used for subsequent cell migration assays.

### Cell migration assay

A 500 µm gap between two sheets of cells was created by removing a two-chamber migration insert (Ibidi, Gräfelfing, Germany) after allowing cells to adhere overnight. Media containing indicated drugs was add at the time of removing the chamber, and time to wound closure was monitored by automated phase-contrast imaging at 15-min intervals.

### Mouse studies

Female C57BL/6 J mice 8–9 weeks of age (Jackson Labs, Bar Harbor, ME) were randomized to either a control (Con, 10 kcal% fat, D12450J, Research Diets, New Brunswick, NJ) or diet-induced obesity (DIO, 60 kcal% fat, sucrose-matched to Con, D12492, Research Diets) diet for 15 weeks. Mice were then randomized to either continue their same diet or be provided the same diet supplemented with 140 ppm sulindac for 5 weeks. This achieves a daily dose of 20 μg/g body weight for control mice, or, when allometrically scaled, roughly 250 mg daily for a 100-kg human [61]. Mice were orthotopically injected with 2 × 10^4^ cells from one of three metastatic cell lines (metM-Wnt^lung^, metM-Wnt^liver^, or E0771) and were monitored to tumor endpoint. Tumor growth was monitored by digital calipers and when tumors in any group reached 1.5 cm in any direction, all mice bearing tumors of the same cell line were euthanized. Tumors and mammary fat pads were excised, weighed, and flash frozen in liquid nitrogen. Lungs were excised, fixed in 10% neutral buffered formalin, and embedded in paraffin. Body composition was determined via quantitative magnetic resonance imaging (Echo Medical Systems, Houston, TX) prior to tumor cell injection. Mice were 33–34 weeks of age at euthanasia.

### Quantification of spontaneous lung metastasis

Four lung sections per mouse were sectioned at 500 μm intervals with individual thickness of 4 μm. Slides were stained with hematoxylin and eosin and evaluated for presence of metastatic cells by a veterinary pathologist blinded to group identity. Data were expressed as a proportion of mice with at least one detectable metastatic lesion.

### Lung colonization

Lung colonization was determined by delivering 5 × 10^5^ E0771 cells by tail vein injection into Con, DIO, ConSul, and DIOSul mice. Similarly to orthotopic tumor models diet-induced obesity was achieved with 15 weeks of high-fat diet feeding, followed by 19 weeks of sulindac treatment before tumor cell injection. Mice were monitored for evidence of labored breathing and euthanized after 4 weeks. Lungs were excised and weighed to determine tumor burden. Mice were 44–45 weeks of age at euthanasia.

### Cell line gene expression microarray analyses

Previously published transcriptomic profiling was access to enable cell line analysis. Raw data were downloaded from GEO accession GSE98703 [35] and analyzed using AltAnalyze [62] to generate raw sample gene expression values, group comparisons, and associated FDRq values. Volcano plots were generated using all relative expression values as the comparison and comparisons with − log10(FDRq) > 2 and log2(fold change) > 1.5 or <−1.5 were considered differentially expressed. Gene Set Enrichment Analysis (GSEA, V4.4.0) was performed using the Hallmark gene sets from the Molecular Signatures Database (MSigDB, V2024.1) [63, 64].

### Bulk tumor transcriptomic profiling gene expression

RNA was isolated from flash-frozen tumor tissue using a TRIzol/column purification method with a Qiagen RNeasy Mini Kit. RNA quality was assessed using a bioanalyzer. Fragmented, biotinylated sense-strand cDNA was generated from total RNA using the *GeneChip™ WT PLUS Reagent Kit* (Applied Biosystems, Carlsbad, CA) according to the manufacturer’s protocol. Hybridization, washing, staining, and scanning were performed on an *Affymetrix GeneTitan™* instrument using the *Clariom™ S Mouse Assay HT* and the *GeneTitan Hybridization, Wash, and Stain Kit for WT Arrays* (Applied Biosystems). Transcriptome data were processed and analyzed with *Transcriptome Analysis Console (TAC) Software v4.0* (Thermo Fisher Scientific). SST-RMA normalized data were used to identify DEG and to perform gene set enrichment analysis (GSEA) using Hallmark gene sets.

### Single cell RNAseq

Approximately 25 mg of freshly excised tumor was dissociated into a single-cell suspension using a cold protease protocol at 4 °C adapted from methods previously described and flow-sorted for live-dead selection and doublet discrimination [65, 66]. Single-cell suspensions were loaded on a 10 × Chromium Controller, and 10 × Genomics 5′ libraries were prepared according to 10 × specifications (10 × Genomics, Pleasanton, CA). Libraries were sequenced on an Illumina NovaSeq 6000 S2 2 × 50 flow cell at the UNC High Throughput Sequencing Facility. Raw data were processed using CellRanger V3.0.0. Filtering, subclustering, and differential gene expression analysis was conducted using Seurat V4.3.0 [67, 68]. Cells with more than 10% mitochondrially encoded transcripts were removed as low quality. TCR sequencing was analyzed using TCRepetoire V3.2.0. GSEA was conducted using Fgsea [69] and visualized using UCell [70]. Over-representation analysis was conducted using clusterProfiler (V3.0.4) [71]. Tumor cell subcluster was determined using GFP expression, filtered based on no *Ptprc* expression, nCount (1,500–50,000), and nFeatures (200–6,000), and clustered using resolution of 0.3 and 30 PCA dimensions. T cell and myeloid subclusters were determined using *Cd3e* and *Itgam* expression respectively, filtered based on no GFP expression, nCount (1,000–25,000), and nFeatures (750–4,500), and clustered using resolution of 0.3 and 12 (T cell) or 15 (myeloid cell) PCA dimensions.

#### Tumor tissue oxylipin analysis

Tumor tissue was homogenized, and lipids were extracted in methanol, centrifuged at 15,000 rcf, and loaded onto an Oasis MAX micro-elution plate (Waters Corp). Samples were then eluted using propanol/acetonitrile (50/50, v/v) containing 5% formic acid. An Acquity I-Class UPLC and Xevo TQ-XS (Waters Corp) triple-quadrupole mass spectrometer operating using multiple reaction monitoring in negative ion mode with argon as the collision gas was used to separate and detect PGE_2_, PGF_2_, 6-keto PGF1a, and 9,10-EpOME. Quantification was expressed as the ratio of sample signal to internal isotopically-labeled standard peak height and normalized to mass of tumor tissue used.

#### Statistical analysis

Graphpad Prism V10.4.2 and R V4.4.2 were used to analyze data. Error is expressed as mean ± SEM. Differences between groups were determined using one-way ANOVA or two-way ANOVA as indicated. Differences between metastatic burden of lungs were determined using pairwise Fisher’s exact test with FDR correction for multiple hypothesis testing. Nonlinear curve-fitting was used to assess in vitro response to indicated compounds. FDRq < 0.05 was considered significant in all analyses.

## Supplementary Information

Supplementary Material 1

## Data Availability

Bulk tumor transcriptomic data are available at GSE294944 and scRNAseq data are available at GSE294999. All other data is contained within the article and its additional files, further details available on request to the corresponding authors (SDH and MFC).
