# Supplementary material for: The nonsteroidal anti-inflammatory drug sulindac reverses obesity-driven immunosuppression and triple-negative breast cancer progression
Source: Breast Cancer Res. 2025 Oct 24;27:186. doi: 10.1186/s13058-025-02134-2 (PMC12551206; doi:10.1186/s13058-025-02134-2)
Supplement: Supplementary file 1 — Supplementary Material 1 [file 13058_2025_2134_MOESM1_ESM.pdf]

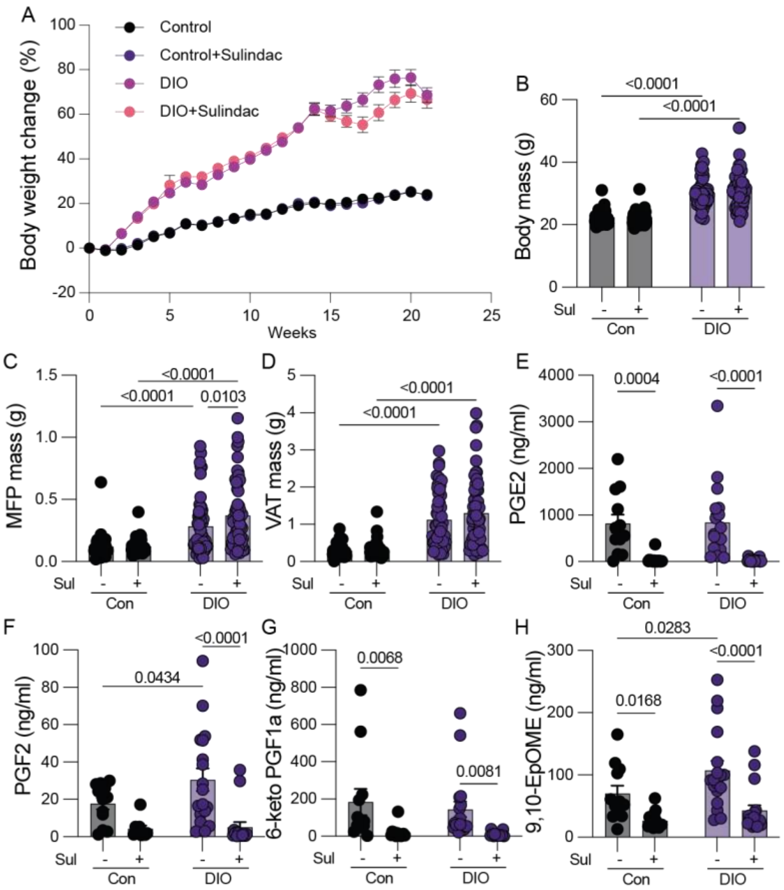

Figure S1: Sulindac supplementation limits PGE_2 production without weight loss

Body weight change (%) of Con and DIO mice before (weeks 0-15) and after (weeks 16-23) exposure to sulindac (n=53-59/group). Terminal body mass (B) (n=51-59), mammary fat pad mass (C) (n=53-59), and visceral fat mass (D) (n=50-58). PGE_2, PGF_2, 6-keto PGF1α, and 9,10-EpOME levels from E0771 tumors (E-H) (n=12-18). Statistical significance determined by two-way ANOVA followed by Šídák's post hoc test (B-H).

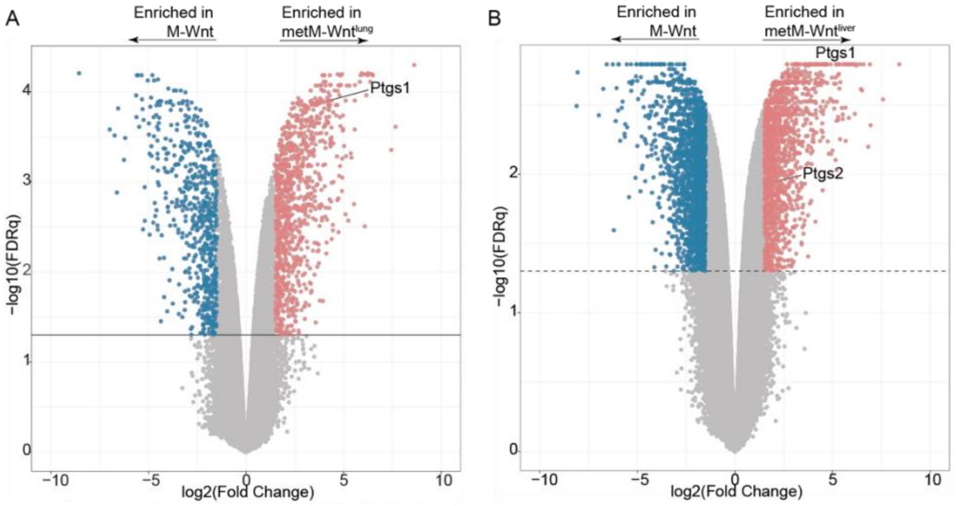

Figure S2: COX expression is elevated in metastatic TNBC subclones

DEG from pairwise comparisons of in vitro transcriptomic profiles of M-Wnt with metM-Wnt^{lung} (A) or metM-Wnt^{liver} cells (n=3/group).

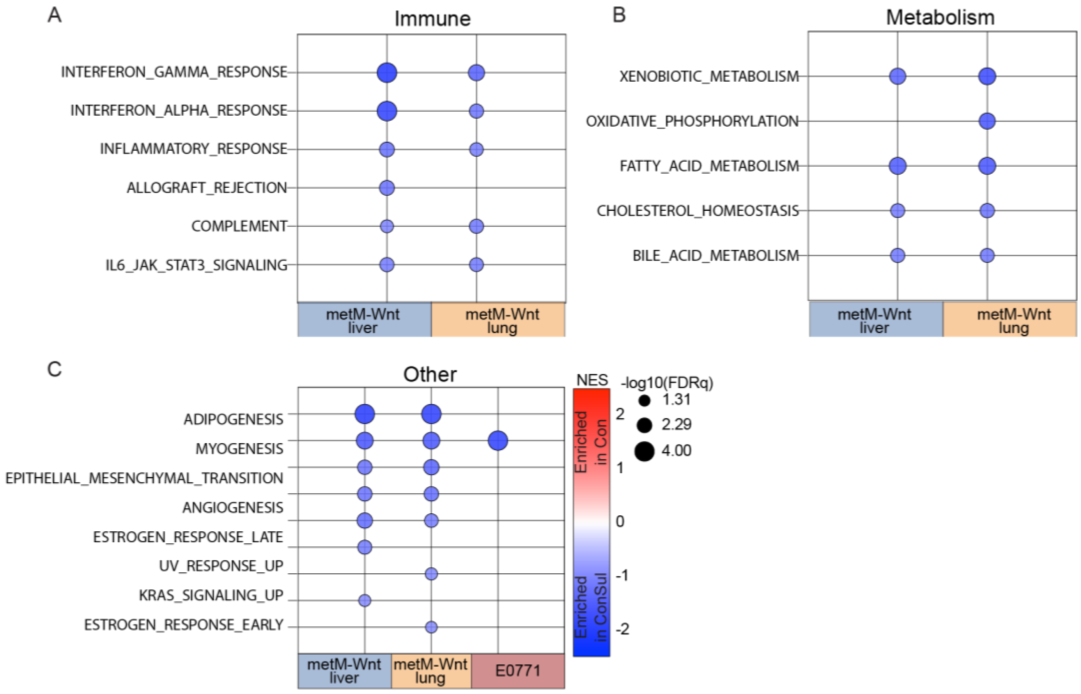

Figure S3: Sulindac supports antitumor immunity in control mice

Significantly enriched GSEA Hallmark gene sets in pairwise comparisons of tumoral transcriptomic profiles of metM-Wnt^{lung}, metM-Wnt^{liver}, and E0771 tumors (n=4-7/group). Transcriptomic profiles for tumors generated from each cell line were subjected to pairwise GSEA comparisons of Con vs. ConSul for immune- (A) and metabolism-related (B), and other gene sets (C). Normalized enrichment score (NES) and -log10 (FDRq) are presented. FDRq <0.05 was considered significant.

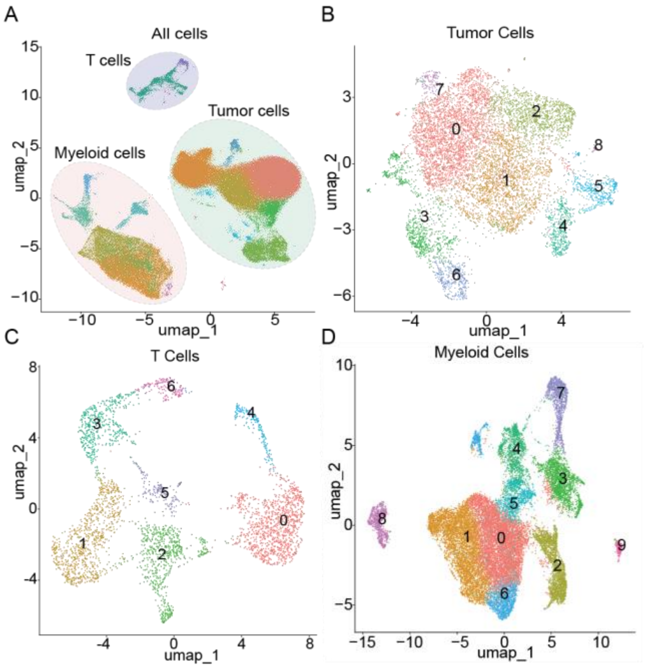

Figure S4: Subclustering approach for scRNAseq analysis

scRNAseq of cells from E0771 tumors were sequentially subclustered based on GFP expression and then immune cell markers (*Cd3e* and *Itgam*). Global UMAP projection (A). Tumor cell subcluster UMAP projection (B). T cell subcluster UMAP projection (C). Myeloid cell subcluster UMAP projection (D).

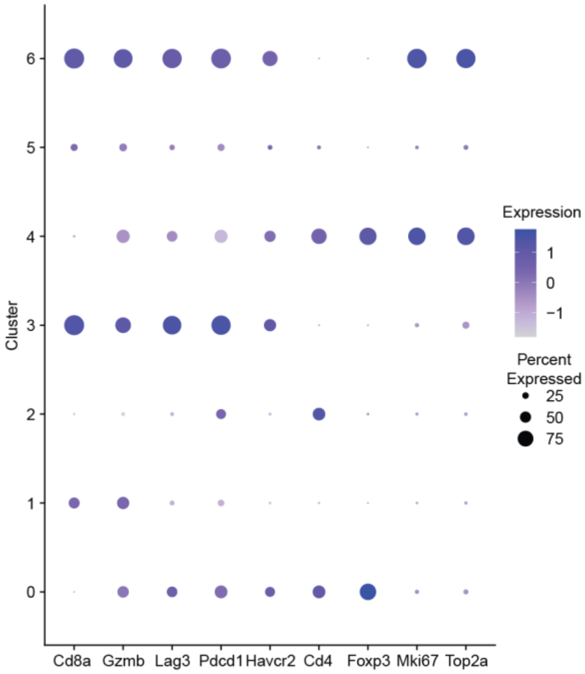

Figure S5: Expression of T cell marker genes

scRNAseq of T cells subclustered based on *Cd3e* expression. Average scaled expression (z-score) and percentage of cells expressing representative marker genes in each cluster.

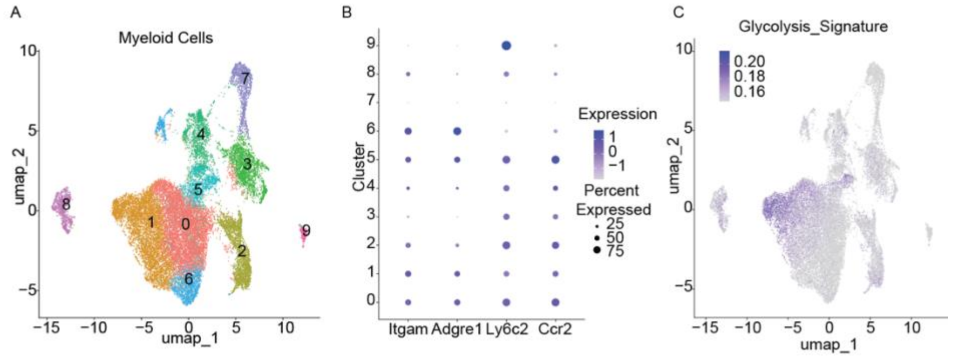

Figure S6: Expression of myeloid cell marker genes

scRNAseq of myeloid cells subclustered based on *Itgam* expression. Average scaled expression (z-score) and percentage of cells expressing the transcript in each cluster are presented. UMAP projection of global myeloid cell population (A). Average scaled expression (z-score) and percentage of cells expressing representative marker genes in each cluster (B). UMAP projection overlaid with glycolysis signature (C).

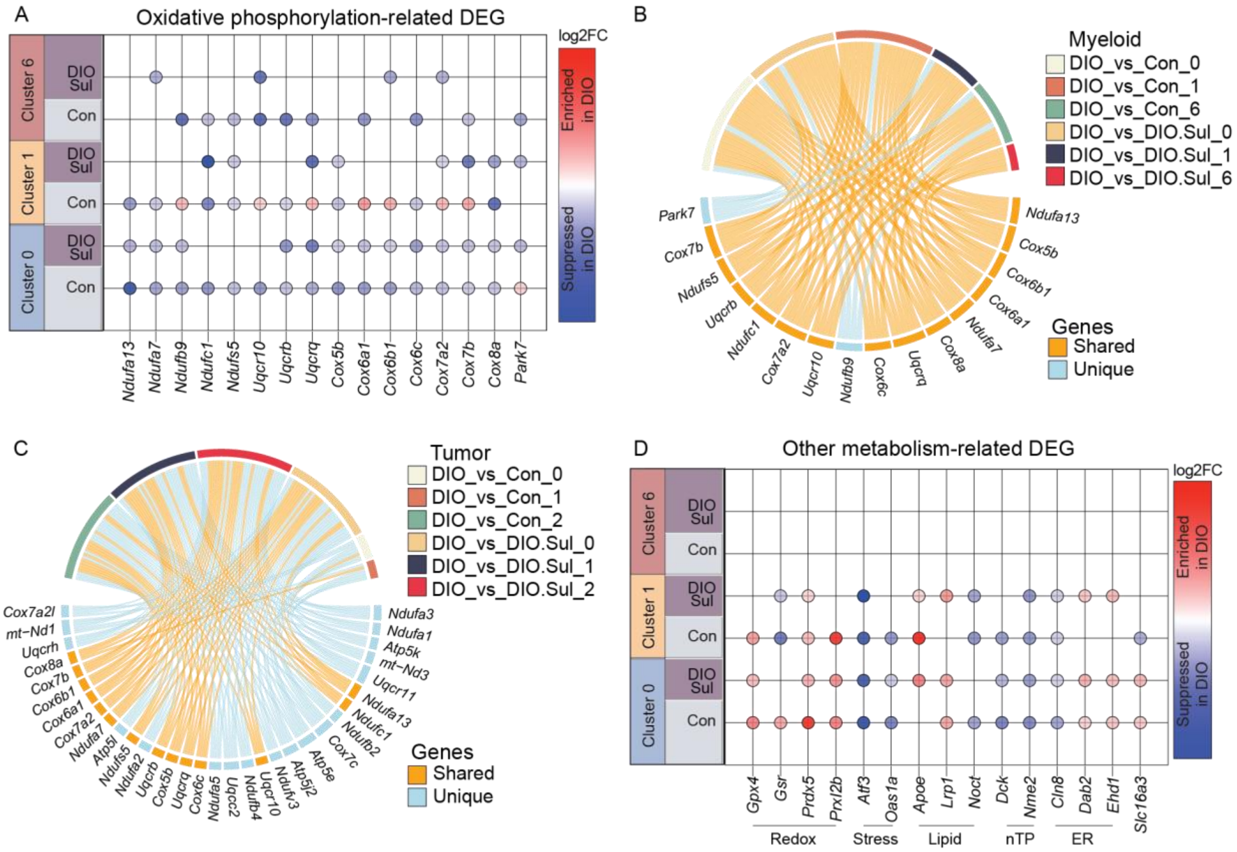

Figure S7: TAM metabolic gene expression altered by obesity is restored by sulindac

Clusterwise analysis of differential expression of metabolic genes between DIO and Con or DIOSul TAM clusters. Oxidative phosphorylation-related genes differentially expressed in at least 3 comparisons (A). Identification of overlap between oxidative phosphorylation-related genes differentially expressed in at least 3 comparisons for both TAMs and tumor cells (B-C). Other metabolism-related genes differentially expressed in at least 3 TAM comparisons (D). FDRq <0.05 was considered significant.
